# Supplementary material for: Empirical methods for controlling false positives and estimating confidence in ChIP-Seq peaks
Source: BMC Bioinformatics. 2008 Dec 5;9:523. doi: 10.1186/1471-2105-9-523 (PMC2628906; doi:10.1186/1471-2105-9-523)
Supplement: Additional File 1 — A variety of html documents from the USeq web site detailing the available applications, their best usage, output file type descriptions, command line menus, etc. [file 1471-2105-9-523-S1.zip › USeqUserGuides/JavaDoc.zip!/JavaDoc/util/gen/package-frame.html]

util.gen


util.gen

Classes 
  
Bin
  
CubicSpline
  
Email
  
FileSize
  
Histogram
  
HTML
  
IO
  
Job
  
LinearRegression
  
MD5
  
MD5Crypt
  
Misc
  
Num
  
Passwords
  
PearsonCorrelation
  
RankedFloatArray
  
RankSample
  
RankSampleIndexComparator
  
RankSampleValueComparator
  
ReadZip
  
SpearmanCorrelation
  
SQL
  
Swing
  
TextFrame
  
XML
